# Generalized Multiscale Entropy Analysis: Application to Quantifying the Complex Volatility of Human Heartbeat Time Series

**DOI:** 10.3390/e17031197

**Published:** 2015-03-12

**Authors:** Madalena D. Costa, Ary L. Goldberger

**Affiliations:** 1 Beth Israel Deaconess Medical Center, Harvard Medical School, Boston, MA 02215, USA; 2 Wyss Institute for Biologically Inspired Engineering at Harvard University, Boston, MA 02215, USA

**Keywords:** aging, complexity, entropy, fractal, heart rate, multiscale entropy, nonlinear dynamics

## Abstract

We introduce a generalization of multiscale entropy (MSE) analysis. The method is termed MSE*_n_*, where the subscript denotes the moment used to coarse-grain a time series. MSE*_μ_*, described previously, uses the mean value (first moment). Here, we focus on ${\text{MSE}}_{\sigma^{2}}$, which uses the second moment, *i.e.*, the variance. ${\text{MSE}}_{\sigma^{2}}$ quantifies the dynamics of the volatility (variance) of a signal over multiple time scales. We use the method to analyze the structure of heartbeat time series. We find that the dynamics of the volatility of heartbeat time series obtained from healthy young subjects is highly complex. Furthermore, we find that the multiscale complexity of the volatility, not only the multiscale complexity of the mean heart rate, degrades with aging and pathology. The “bursty” behavior of the dynamics may be related to intermittency in energy and information flows, as part of multiscale cycles of activation and recovery. Generalized MSE may also be useful in quantifying the dynamical properties of other physiologic and of non-physiologic time series.

*I have ideas and reasons*,

*Know theories in all their parts*,

*And never reach the hearts*.

- Fernando Pessoa (translation)

## 1. Introduction

The trajectories of the human heartbeat constitute an object of scientific, as well as literary, inquiry. Fluctuations in cardiac interbeat intervals (often termed heart rate variability) are regulated by the autonomic (involuntary) nervous system, which plays a fundamental role in integrative physiologic control. Heart rate time series, therefore, provide a unique window into the status of the cardiovascular system in health and disease and, more broadly, into the entirety of physiologic function [1].

From a dynamical perspective, cardiac interbeat interval time series pose major challenges to quantitative analysis. These signals are typically non-stationary, non-linear and exhibit complex fluctuation patterns over a wide range of time scales. The multiscale entropy method (MSE) [2] was developed to probe the information content of this class of signals. Using this method, we [3–7] have provided evidence that the complexity of cardiac interbeat interval time series decreases with aging and disease.

The MSE method employs an entropy measure to quantify the degree of unpredictability of time series derived from the original signal by an operation called coarse-graining. This operation consists of dividing the original signal ({*x_i_*}, 1 ≤ *i* ≤ *N*) into non-overlapping segments of equal length (*τ*) and calculating the mean value of the data points in each of these segments. The process is repeated for a range of window lengths, *i.e.*, scales. Therefore, the derived time series are coarse-grained outputs of the dynamical system. The complexity index, based on the work of Zhang [8], is defined as the sum of the entropies computed for different scales, *i.e.*, at different levels of resolution of the signal. Such a complexity measure yields low values for both highly regular (e.g., periodic signals) and highly irregular (uncorrelated random noise) signals and is near maximum for signals with 1/f, long-range correlations. These observations are consistent with the notion that systems at either extremes of the “entropy spectrum” (*i.e.*, having essentially zero entropy or being maximally entropic) are not complex [2–4].

Intuitively, by quantifying the level of disorder at all relevant levels of resolution of a signal, the MSE method yields a measure of its complexity. An unaddressed question is whether the coarse-graining procedure, itself, which uses a single property of the data, *i.e.*, the mean value, to derive copies of the original signal at different levels of resolution, discards important information whose quantification could enhance our understanding of the underlying processes. To help address this question, we generalize the MSE method to a family of statistics (MSE*_n_*) by using different moments (*n*) of the distribution of a random variable to coarse-grain the original time series. The previously described MSE method is termed MSE*_μ_*, where *μ* refers to the mean (first moment).

Here, we implement the ${\text{MSE}}_{\sigma^{2}}$ method, which uses the variance (second moment) to coarse-grain the signals. We apply this new method to cardiac interbeat interval time series from healthy young and older subjects, and patients with congestive (chronic) heart failure [2,3,9]. This syndrome (especially the systolic type) develops when cardiac output is not sufficient to meet metabolic requirements, despite high ventricular filling pressures [10]. It represents one of the most extreme manifestations of loss of adaptiveness. The resulting derangements alter autonomic function and, consequently, heart rate dynamics. To a lesser extent, the aging process also decreases adaptiveness and the complexity of cardiac interbeat interval dynamics [2–5,11].

We test the hypothesis that, under baseline (“free-running”) conditions, the heartbeat volatility time series from healthy young subjects are more complex than those of healthy older subjects, which, in turn, are more complex than those from patients with heart failure.

## 2. Methods

Consider a time series ({*x_i_*}, 1 *≤ i ≤ N*). MSE*_n_* is computed as follows. First, the original signal is divided into non-overlapping segments of length *τ*. Second, a selected moment is estimated for the data in each of these segments to derive the coarse-grained time series at scale *τ*. Here, we focus solely on the second moment using an unbiased estimator $\left(\sigma^{2}=1/(N-1)\sum_{i=1}^{N}{\left(x_{i}-x\right)}^{2}\right)$ of variance. Third, a measure of entropy, sample entropy [12], is calculated for each coarse-grained time series. Fourth, a complexity index is derived by adding the entropy values for a selected range of scales.

We analyzed cardiac interval (RR) time series derived from approximately 24 h continuous electrocardiographic (ECG) Holter monitor recordings of 26 ostensibly healthy young subjects (13 men and 13 women, aged (mean ± SD) 35±7.4, range 20–50 years), 46 ostensibly healthy older subjects (22 men and 24 women, aged 65 ± 4.0, range 58–76) and 43 patients with moderate to severe congestive heart failure syndrome of various etiologies (28 men and 15 women, aged 55.5 ± 11.4 years, range 22–78) [3]. The ECG recordings from healthy subjects were sampled at 128 Hz. Fourteen recordings from patients with heart failure were sampled at 250 Hz and 29 at 128 Hz.

Datasets were filtered to exclude artifacts, premature ventricular complexes, and missed beat detections. The algorithm is available at http://www.physionet.org/physiotools/apdet/apdet-1.0/filt.c [9]. Briefly, the central point of a moving window of length *l* is excluded if it lies outside the interval $\left[\bar{x}\pm a{\bar{x}}_{i}\right]$, where ${\bar{x}}_{i}$ represents the average of the data points in that moving window, calculated excluding the central point, and *a* is a positive number ≤ 1. Here, we used *l* = 41 and *a* = 0.2. For the calculation of the complexity index we selected scales 10 to 100. For the calculation of SampEn we used *m* = 2 and *r* = 0.5% of the original time series’ standard deviations. Note that MSE*_μ_* analysis of the same data [3] was performed using *m* = 2 and *r* = 15% of the original time series’ standard deviations. The difference in the choice of the *r* values was due to the fact that the amplitudes of the variance coarse-grained time series are much smaller than those of the mean coarse-grained time series.

## 3. Results

**Figure 1:**
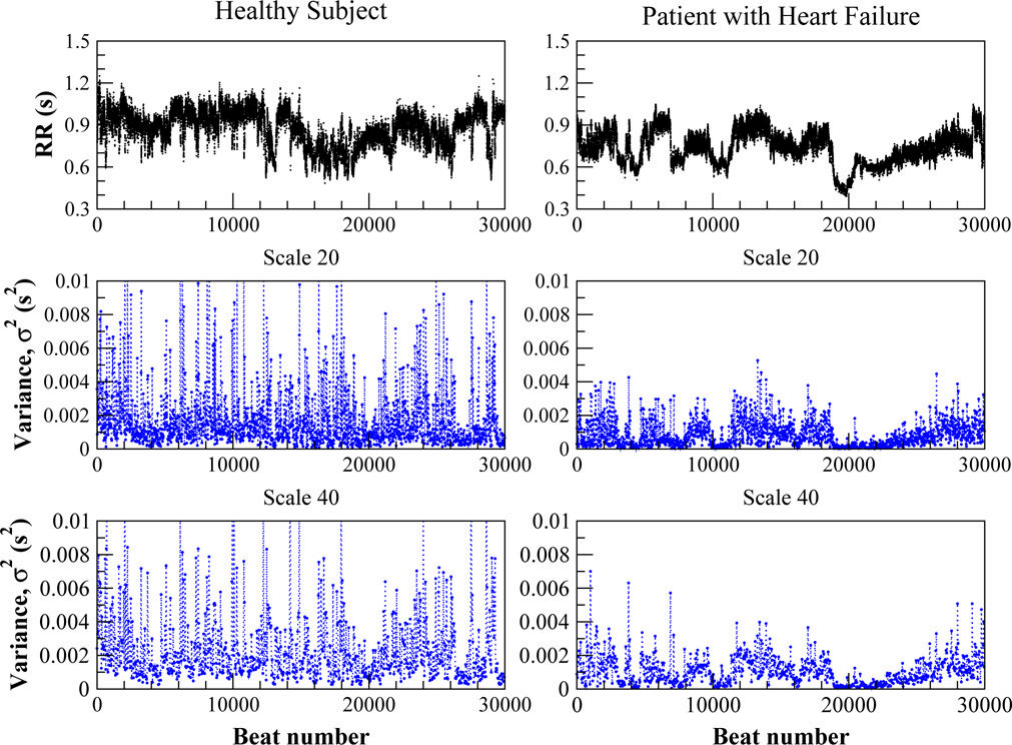
Top: Cardiac interbeat interval (RR) time series from a healthy 20 year-old subject (left) and a 53 year-old patient with congestive heart failure (right). Middle and bottom: Variance of the RR interval time series calculated in a 20 (middle) and 40 (bottom) data point moving window. The horizontal axes are the same for all plots.

Figure 1 shows the RR interval time series from a healthy subject and from a patient with congestive heart failure (top panel), and the corresponding variance derived coarse-grained time series for scales 20 and 40 (middle and lower panels). The latter panels show complex fluctuation patterns with higher amplitude in the case of the healthy subject. We note that the structure of the fluctuations appears to be preserved with re-scaling in both the healthy and pathologic cases.

**Figure 2:**
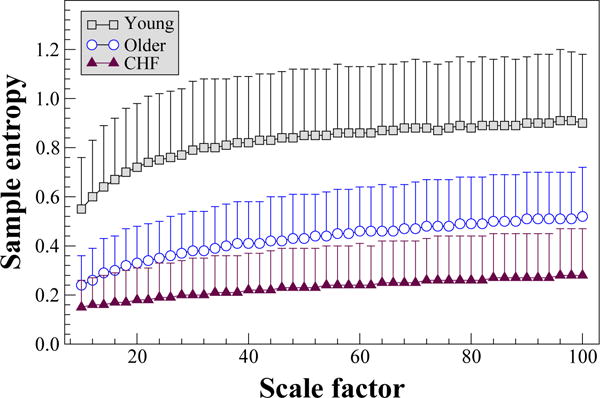
Multiscale entropy ${\text{(MSE)}}_{\sigma^{2}}$ analysis of cardiac interbeat interval time series from 26 healthy young, 46 healthy older subjects and 43 patients with congestive heart failure (CHF). The time series were derived from 24 h Holter monitoring recordings. Parameters for calculating sample entropy: *m* = 2, *r* = .5% of the original time series’ standard deviations. MSE*_μ_* analysis of the same time series were presented in [3]. The symbols and the error bars represent mean and standard deviation, respectively. The time series are available at www.physionet.org/physiobank/database/, under nsrdb, nsr2db, chfdb and chf2db.

Figure 2 shows the results of the ${\text{MSE}}_{\sigma^{2}}$ analysis of RR interval time series from three groups, comprising health young and older subjects, and patients with chronic heart failure syndrome. The mean and standard deviation values of the complexity indices for the young, older and heart failure groups were 75.2 ± 24.3, 39.0 ± 15.7 and 20.9 ± 14.1, respectively. The complexity indices of healthy young subjects were significantly higher than those of healthy older subjects (p < 0.0001, two-tail Mann-Whitney test) and of patients with heart failure (p < 0.0001). In addition, the complexity indices of the healthy older subjects were significantly higher than those of the heart failure patients (p < 0.0001). These intergroup differences were confirmed using a fixed *r* value (0.0002 s^2^) for the computation of sample entropy as described in [13], and using quadratic entropy [14] in place of sample entropy. Both of these methods mitigate the impact of outlier values on entropy estimates. In addition, comparable intergroup differences to those presented here were obtained using an *r* value that is a percentage of one of the first variance coarse-grained time series.

## 4. Discussion

This paper introduces a generalization of the multiscale entropy (MSE*_n_*) method based on employing different moments to coarse-grain a time series. The original method (MSE*_μ_*) quantifies the complexity of fluctuations in the local mean value of a variable. Here, we focus on ${\text{MSE}}_{\sigma^{2}}$, which quantifies the dynamical properties of volatility over multiple time scales. We use the variance to derive the coarse-grained time series and an entropy measure to probe their structure. We applied this technique to heartbeat time series from healthy young and older adults, and patients with congestive heart failure syndrome. Our method reveals that human heartbeat volatility time series (Figure 1) exhibit complex bursting behaviors over a wide range of time scales.

Traditional fractal analysis [15] methods essentially quantify how the mean amplitude of coarse-grained time series derived using standard deviation changes with scale factor, but do not probe the temporal structure of the signal at a given time scale. In contrast, ${\text{MSE}}_{\sigma^{2}}$ quantifies the dynamics of each of the variance coarse-grained time series using an entropy measure.

A key physiologic finding is that the multiscale complexity of the volatility, not only of the mean heart rate [2–4], degrades with aging and pathology. The mechanism of healthy heartbeat volatility remains to be established. We speculate that this finding relates to bioenergetic fluxes. Cardiovascular function requires electromechanical pulses of activation and recovery (depolarization/repolarization and systole/diastole). The concept of the heartbeat pulse, which is definitional to basic physiology and clinical medicine, may deserve broader consideration as a multiscale, not just a single-scale, phenomenon.

Mathematical models purporting to capture the dynamics of healthy heartbeat variability should account for the observed multiscale volatility and for its degradation with aging and disease. Our method also opens up inquiries into the use of MSE methodology to probe properties of the signals related to higher moments (e.g., the third moment, *i.e.*, skewness) [16]. We will explore analytic analyses and numerical simulation studies of MSE*_n_*, as well as chronobiologic effects, in subsequent publications. Studies of the relationship between MSE*_n_* and multifractal and related measures [18–21] may also be productive. Furthermore, our method can be used to probe the properties of time series from other physiologic systems with known pulsatile behavior (e.g, speech, neural recordings, hormonal fluctuations, etc) and to test whether the complexity of physiologic volatility also degrades in different pathologies, including cancer. A requirement for such analyses is that the signals be recorded for sufficient duration and with high enough temporal resolution to afford adequate statistical representation of behavior across a relevant range of time scales. The MSE implementations introduced in [17] may be useful, especially for the analysis of relatively short time series. Finally, the MSE*_n_* analysis of non-physiologic time series, e.g., econometric, may be of interest.
